# An open-source toolbox for automated phenotyping of mice in behavioral tasks

**DOI:** 10.3389/fnbeh.2014.00349

**Published:** 2014-10-08

**Authors:** Tapan P. Patel, David M. Gullotti, Pepe Hernandez, W. Timothy O'Brien, Bruce P. Capehart, Barclay Morrison, Cameron Bass, James E. Eberwine, Ted Abel, David F. Meaney

**Affiliations:** ^1^Department of Bioengineering, University of PennsylvaniaPhiladelphia, PA, USA; ^2^Department of Biology, University of PennsylvaniaPhiladelphia, PA, USA; ^3^Department of Neuroscience, University of PennsylvaniaPhiladelphia, PA, USA; ^4^Department of Psychiatry and Behavioral Sciences, Duke UniversityDurham, NC, USA; ^5^Department of Biomedical Engineering, Columbia UniversityNew York, NY, USA; ^6^Department of Biomedical Engineering, Duke UniversityDurham, NC, USA; ^7^Department of Pharmacology, University of PennsylvaniaPhiladelphia, PA, USA; ^8^Department of Neurosurgery, University of PennsylvaniaPhiladelphia, PA, USA

**Keywords:** automated behavior, blast-induced traumatic brain injury, Elk-1 knockout, spatial object recognition, social interaction, Barnes maze

## Abstract

Classifying behavior patterns in mouse models of neurological, psychiatric and neurodevelopmental disorders is critical for understanding disease causality and treatment. However, complete characterization of behavior is time-intensive, prone to subjective scoring, and often requires specialized equipment. Although several reports describe automated home-cage monitoring and individual task scoring methods, we report the first open source, comprehensive toolbox for automating the scoring of several common behavior tasks used by the neuroscience community. We show this new toolbox is robust and achieves equal or better consistency when compared to manual scoring methods. We use this toolbox to study the alterations in behavior that occur following blast-induced traumatic brain injury (bTBI), and study if these behavior patterns are altered following genetic deletion of the transcription factor Ets-like kinase 1 (Elk-1). Due to the role of Elk-1 in neuronal survival and proposed role in synaptic plasticity, we hypothesized that Elk-1 deletion would improve some neurobehavioral deficits, while impairing others, following blast exposure. In Elk-1 knockout (KO) animals, deficits in open field, spatial object recognition (SOR) and elevated zero maze performance after blast exposure disappeared, while new significant deficits appeared in spatial and associative memory. These are the first data suggesting a molecular mediator of anxiety deficits following bTBI, and represent the utility of the broad screening tool we developed. More broadly, we envision this open-source toolbox will provide a more consistent and rapid analysis of behavior across many neurological diseases, promoting the rapid discovery of novel pathways mediating disease progression and treatment.

## Introduction

An increasing number of behavioral assays are available to the neuroscience community for identifying a phenotype in mouse behavioral studies. Many of these behavioral tasks are linked to one or more neuroanatomic substrates (Phillips and Ledoux, 1992; Broadbent et al., 2004; Balderas et al., 2008; Barker and Warburton, 2011). As such, rapidly defining a behavioral phenotype could bridge the gap between changes in brain structure and the advancement of new therapies for treating neurological diseases.

Key bottlenecks limit behavior phenotyping across laboratories. Many tests use time-intensive manual scoring techniques susceptible to inter-operator variability, leading to poor reproducibility within and across research groups. Moreover, manual tracking methods do not provide an opportunity to explore or “re-mine” data not collected during the initial scoring. Although automated activity monitoring methods exist to increase the speed of analysis and reduce variability, the methods are either proprietary, not robust, or rely on specialized, expensive equipment not widely accessible to the research community. Similarly, automated scoring methods currently do not allow adjustments to either improve the accuracy or extend the analysis of several common behavior tests.

In parallel, the analytical framework to extract the significant, unique behavior patterns across experimental groups needs better definition. Rather than evaluating behavioral tasks independently using traditional parametric or nonparametric statistical tests, a single consolidated analysis may identify significant groupings, or patterns, of behaviors (Markow and Hanson, 1981; Vekovischeva et al., 2007). The consolidated analysis of several tasks will become even more important as we increase our ability to automate task scoring, and this systems-level analysis would prove increasingly valuable to prospectively identify brain areas most affected by the genetic manipulation or disease condition.

Recognizing the benefits of an automated system, the neuroscience community has developed many different methods to automate the phenotyping of animals in their home-cage (Tamborini et al., 1989; Casadesus et al., 2001; Tang et al., 2002; Millecamps et al., 2005; Tang and Sanford, 2005; Chen et al., 2006; Steele et al., 2007; Bonasera et al., 2008; Goulding et al., 2008). In contrast, automation of video recordings of *task-related* experiments lags behind. Existing home-cage software, including most recent machine learning (Kabra et al., 2013) or computer vision (Jhuang et al., 2010) based methods cannot be applied to score task-experiments, partly because these methods are primarily designed to classify the way in which a mouse's body deforms over small time intervals and assign behavioral labels such as rearing, grooming, or sitting. Scoring task-related experiments requires an entirely different approach based on the temporal evolution of an animal's *interactions* with the environment [e.g., exploration of objects in spatial object recognition (SOR) or social interaction] or by the *choices* the animal makes (e.g., entry into different regions of an arena as in Y-Maze, place-preference, etc.). Only recently have tools emerged to score some common tasks, or, more generally, a more general purpose tools to develop automated scoring functions [e.g., Janelia Automatic Animal Behavior Annotator (JAABA); Kabra et al., 2013].

We now significantly extend the repertoire of computerized methods for scoring video recordings of many behavior tasks that span tests of anxiety, cognition, learning, and memory. These include fear conditioning, open field, zero-maze, Y-maze, plus-maze, T-maze, Barnes maze, place preference, SOR, novel object recognition (NOR), and two- or three-chamber social interaction. We overcome the limitations of existing methods that either required inking part of the animal for automatically identifying body landmarks (Rutten et al., 2008) or required specialized equipment to monitor activity. For each behavior task, we use this new toolbox to automatically compute performance metrics that are commonly scored manually and achieved equal or better consistency compared to inter-observer variability. In addition, we introduce novel fine-grained measurements of task performance that are not available through manual scoring.

We employ some of these tools and a systems-level analysis to evaluate how the aggregate behavior of animals changes with a genetic and/or experimental manipulation. This *auto*mated pheno*typing* of behavior, or autotyping, reveals a novel behavior pattern for a mouse model of blast-induced traumatic brain injury (bTBI). We hypothesized that, due to its role in neuronal survival and proposed role in synaptic plasticity (Sharma et al., 2010; Besnard et al., 2011; Morris et al., 2013), the genetic deletion of transcription factor, Ets-like kinase 1 (Elk-1), would ameliorate some, but not all, behavior impairments of bTBI. Indeed, we find that bTBI increases anxiety-like behavior in wild-type mice and this effect is significantly reduced in Elk-1 knockout (KO) animals.

## Methods

### Subjects

All animal studies were conducted according to NIH guidelines and were approved by the University of Pennsylvania's Institutional Animal Care and Use Committee (IACUC). We studied the behavioral effects of bTBI using an Elk-1 KO mouse (Cesari et al., 2004) and wild-type littermate (WTLM) mice.

### Blast-induced traumatic brain injury (TBI)

We used a shock-tube to generate a fully developed shock wave within an aluminum tube. The animal was placed 16-mm from the exit of the tube, and experienced a typical blast overpressure loading—a rapid rise in pressure (40 μs) followed by a slightly longer pressure decay (0.615 ms) (Gullotti et al., 2014). For all experiments, we used blast input conditions (peak overpressure: 215 kPa, duration: 0.65 ms) that, when averaged across three pressure transducers placed along the periphery of the exit of the tube, varied less than 5% across all animals tested, and caused an immediate impairment in righting reflex. Once animals recovered their righting reflex, they were returned to a warmed recovery cage.

### Movement detection, tracking, and orientation overview

Several simple observations from the video record were automated: (1) determining whether the animal was moving and classifying the type of motion (goal-directed or exploratory), (2) determining the absolute location of the animal in an arena and relative to other objects, (3) identifying several landmarks on the animal's body, and (4) determining the animal's gaze direction and body curvature. These movement classifiers were key for determining an automated score for a given test. All algorithms described below are implemented in MATLAB (MathWorks). The source-code, detailed user guide, and sample experiment videos are freely available on www.seas.upenn.edu/~molneuro/autotyping.html.

### Object tracking and detection of interactions with the environment

We automated the process for determining the precise location of an animal and time spent interacting with an object or within a region of interest (ROI). Traditionally, automated identification of interaction has been a difficult task. A common method uses photobeam crossings in an open field to determine the location of an animal in an arena. However, this method requires the user to predetermine areas of interaction, requires calibration of additional monitoring equipment and the spatial resolution is limited to the density of photobeams. To our knowledge, the only other open-source automated software for object interaction requires inking the mouse's tail to denote a starting point and iteratively searches for position of the nose via multiple line fittings (Rutten et al., 2008), a process that can easily create cumulative errors. In our experience, proprietary software (e.g., Clever Systems) often suffered from this limitation, restricting its utility. Our algorithm consisted of segmenting the mouse in the image; determining locations of head, tail, and centroid; determining the direction of gaze; extrapolating whether the mouse's line of site crosses an ROI; and assigning a label (interacting or not interacting) to each frame.

**Figure 1:**
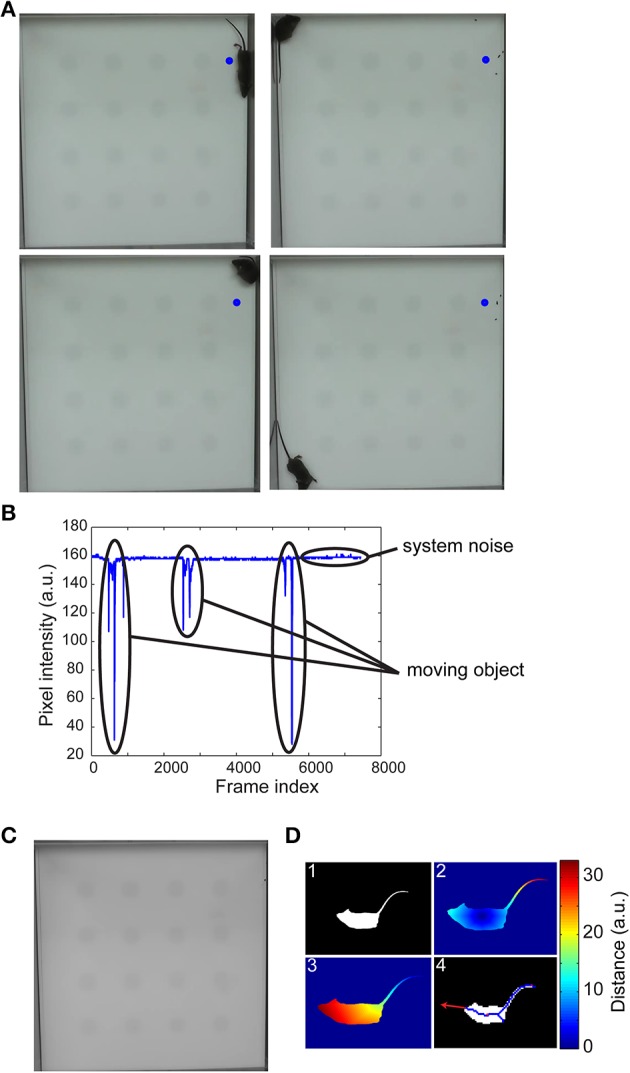
**Background estimation, segmentation, and detection of the head**. Four randomly selected frames of a 10-min video of an open-field experiment **(A)** shows the different locations of the mouse in the arena. The pixel intensity variation at the center of the blue circle illustrates sparse variations from baseline intensity due to a moving object **(B)**. The first mode of pixel intensity histogram at each pixel location accurately estimated the background scene **(C)**. The mouse was segmented by thresholding a background subtracted image **(D1)** and the centroid, tail **(D2)**, and head **(D3)** coordinates determined via a geodesic distance transform (see main text for details). A vector from the centroid to head or extrapolation of the medial axis provided gaze direction **(D4)**.

Segmentation was accomplished by background subtraction. In selecting an efficient and robust algorithm for estimating the background, we note that typical object interaction experiments are short in duration, have relatively constant (perhaps uneven) illumination, steady background geometry throughout the experiment and have minimal shadowing or hardware motion artifacts (i.e., camera is held in position). If there are no moving objects in the scene and no variations in illumination, then for each pixel location, the intensity values along the temporal axis should be constant; however, moving objects or system noise cause pixel intensity to vary from a constant value. Since the moving objects appear only in a small number of images at any pixel location, an estimate of the background was obtained as the main mode of the underlying distribution along the temporal axis for each pixel location (Figures 1A–C). Estimating the background scene was accomplished in under 1 min on a standard workstation with an Intel i940 processor and 6 GB RAM.

The centroid of the moving segmented object (mouse) and the coordinates of the nose and tail are determined via geodesic distance transform (Figure 1D). We note that the mouse's anatomy is such that the tip of the tail is the farthest geodesic distance from the centroid and its nose is the farthest geodesic distance from the tail. To determine the directions of mouse's gaze, we could either draw a vector from the centroid to the nose coordinates or skeletonize the segmented image and fit a line to points near the head. Both approaches were equally effective in identifying mouse's gaze. Commercial systems were not sufficiently robust in consistently detecting these landmarks, virtually eliminating their usefulness especially in a high-throughput setting.

**Figure 2:**
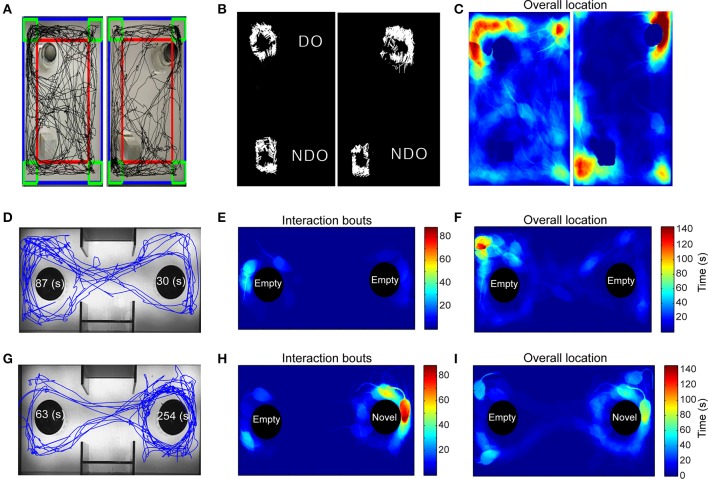
**Application of automated algorithm for scoring “interaction” tasks. (A–C)** Response to novel objects and spatial novelty. **(A)** The path traveled (black) by the mouse during the first exposure session to the objects (left) and during the second exposure where one of the objects is displaced (right). **(B)** Exploration of the displaced and non-displaced objects represented by white lines that denote the angle of approach and the number of exploratory bouts. **(C)** Heat-map representing the mouse position in the experimental arena, red = more time, blue = less time. **(D–I)** Analysis of a social interaction experiment shows path traveled **(D)** and non-biased exploratory bouts **(E)** between the two non-social objects. Majority of the time is spent in a corner **(F)**. After the introduction of a novel mouse in the right chamber, the test mouse demonstrates significant greater preference for the novel mouse-containing object over the empty object **(G**–**I)**.

The overall trajectory of the mouse in an experimental arena was visualized by plotting its centroid coordinates (Figure 2A). The total distance traveled or the amount of time spent interacting with an object across multiple exposures to the same arena are common measures of habituation (Vianna et al., 2000), one of the most elementary nonassociative learning tasks in rodents. Our automated tracking computes this directly in real-time, and also allowed us to plot the angle of approach during each bout of exploration of an object, possibly providing a novel method to examine biases (Figure 2B). In our implementation, users have the flexibility to draw arbitrary number of ROIs denoting objects of potential interaction. An immediate advantage of this flexible ROI assignment appears for the SOR task, where we gain the ability to determine if the mouse acquired spatial memory via drawing a phantom ROI around what used to be the displaced object. Additionally, a heat-map plot of the mouse position during the test facilitates high-throughput characterization of behavior through novel pattern recognition or machine learning algorithms (Figure 2C). The algorithm for detecting interaction with an object is also useful for measuring social interactions (Figures 2D–I).

### Application to automated scoring of tasks

The modular implementation allowed us to extend our methodology for analyzing many neurobehavior tasks. A complete list of behavior tasks and their respective performance metrics that are automatically derived are provided in Table 1. All behavior experiments were videotaped using a securely mounted overhead camera (Logitech C270HD). Social interaction experiments were performed in dark lighting condition and were recorded with a Sony DCR-SR60 camcorder. Video duration varied depending on behavior experiment, ranging from 2 to 30 min. The autotyping software is able to process videos encoded in most widely-used file formats, including .wmv, .avi, .mpg, .mp4, and .mov.

**Table 1:** **Automated scoring of behavior tasks**.

**Behavior test**	**Performance metric**
**Open field**	Thigmotaxis
	Total distance traveled
	Time spent walking, sitting, exploring
	Entries into the center of the arena
**Fear conditioning**	Total freezing time (test session)
	Freezing time immediately after foot shock (training session)
**Elevated zero maze**	Latency to first exit
	Time in open/walled regions
	Total distance traveled
	Risk assessment
**Y-maze, T-maze**	Time spent and distance traveled in each of 3 arms
	Number of spontaneous transitions and total transitions
	Conditional probability of transitions between arms
	Instantaneous speed
**Barnes maze**	Latency to escape
	Number of errors
	Path length to the escape box
	Number and duration of nosepokes
	Strategy to escape—random, systematic, or spatial
**Spatial/noval object recognition**	Time spent interacting with each object
	Direction of approach for each interaction bout
	Spatial extent of object exploration—uniform or one-sided
	Distance, speed and exploratory tendencies
**Social interaction**	Interaction time with inanimate object vs. animate object
	Visual gaze (direction) of exploration

### Spatial object recognition

On the day of training, mice were placed in the training arena for a total of 10-min session. The first session consisted of context habituation without objects in the arena. During the next 3 sessions, mice were allowed to explore the arena with two distinct objects (a glass bottle and a metal tower). Each session lasted 10 min. Testing occurred 24 h after the four training sessions in which one of the two objects was displaced. To analyze these tests, we determined the location and visual field of the mouse during the test procedure. The user defined an ROI for each object in the arena, and the software computed the fraction time (% of total) the animal was interacting with the ROI. During each bout of interaction, the instantaneous direction of gaze was also recorded to determine whether there were direction-approach biases (Figure 2B). For example, the software permits measurement of the interaction time with different sides of the object facing the center, walls or corners of an arena. This level of analysis can be informative for models of autism in which gaze aversion or avoidance is a prominent phenotype (Clifford et al., 2007; Defensor et al., 2011). The mouse's preference for the displaced object over the non-displaced object was measured for all sessions. Video S1 demonstrates real-time tracking and scoring of a SOR experiment.

### Social interaction

A three-chamber test was used to analyze animal's sociability and preference for social novelty. Animals are placed into the middle chamber and allowed to habituate to the arena, containing empty objects in the left and right chambers. In the second trial, a novel mouse is introduced into either the left or right chambers. The test animal's preference for the novel mouse is a measure of sociability. To analyze, we defined two separate ROIs that contain either an inanimate object or a novel mouse. Similar to SOR, we determined the interaction time for both ROIs, the approach angle during each bout of interaction, and distance traveled. Heat-map indicating cumulative time spent in different parts of the sociability apparatus is especially useful to visually inspect preferences between novel objects and novel mice (Figures 2D–I).

### Open field test

**Figure 3:**
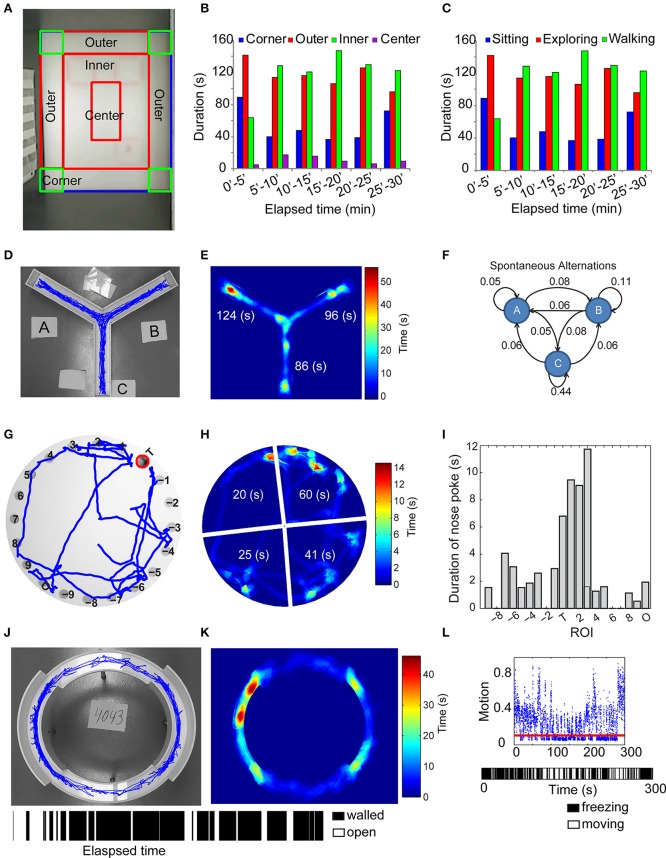
**Automated analysis of several maze-related tasks. (A–C)** Automated tracking **(A)** and measurement of the time spent in different regions of the open-field in any 5-min interval **(B)**, along with the time spent walking, exploring and sitting **(C)**. In Y-maze, the trajectory of the test mouse **(D)**, the amount of time spent in each of the 3 arms **(E)**, denoted as “A,” “B,” “C,” and the relative fraction of transitions between each of the three arms **(F)** are determined as metrics of spatial memory. A standard Barnes-maze consists of 20 circular holes, one of which is the escape box. The 20 holes are automatically identified using pixel intensity gradient and numbered such that the escape box or “target” is denoted “T,” the hole opposite to the escape box denoted “O” and the remaining holes numbered 1–9 and -1 to -9 in clockwise and counterclockwise directions relative to the escape box **(G)**. The latency to escape box and the amount of time spent in each of four quadrants (denied as wedge-shaped areas encompassing sets of five holes) are recorded **(H)**, along with the total number and duration of nosepokes in each of the 20 holes **(I)**. In elevated zero-maze, the mouse is placed in a walled-region and the latency to escape and the amount of time spent in walled or open regions of the maze are measured [**(J,K)** bar graph with alternating black and white stripes indicate the location of the mouse in walled (black) or open (white) regions of the maze as a function of time]. **(L)** (Top) Discrimination of motion from freezing events using an estimate of camera noise (red line). (Bottom) Time strip showing bout length of freezing behavior (white space) relative to movement bouts (black space) in fear conditioning.

Individual mice were released in the corner of a rectangular (30 × 40 cm) open field arena. Mice were left undisturbed and videotaped with a camera mounted on the ceiling above the center of the open field arena for 30 min. At the end of testing, mice were returned to their home cage. We automatically partitioned the video arena into outer periphery, inner, and center region and four corner quadrants. Using the automated tracking of the mouse centroid, the software computed the amount of time spent and the distance traveled in these subdivisions (Figures 3A,B). The ambulation data was further categorized as walking (straight and relatively fast locomotor activity), exploring (non-straight line path locomotion performed at a relatively slow speed), or sitting (non-locomotion for at least 3 s) (Figure 3C) (Choleris et al., 2001).

### Y-maze task

Mice were placed in the center of a Y-shaped maze and allowed to freely navigate throughout the maze. We recorded the motion of the animal during the navigation phase for 8 min. The user identified the maze arms in the video and our motion-tracking algorithm allowed us to detect animal position throughout the testing period (Figure 3D). The number of crossings into each of the three arms of the Y-maze was recorded in real time. The final measurements from the Y-maze were the number of spontaneous alternations, the time spent in the central portion and the three arms of the maze (Figure 3E), and the relative fraction of crossings into each arm (Figure 3F). Video S2 demonstrates real-time tracking and spontaneous alternations between arms of the Y-maze.

### Barnes maze

Animals were placed in the center of a Barnes maze containing 20 separate holes, one of which contained an escape box. Over repeated trails, we recorded the motion of the animal as it explored the environment and found the correct escape hole. To automate this process, we identified the target hole and labeled it “T,” identified the hole opposite target “O” and numbered the rest as 1–9 or −1 to −9. Using motion tracking algorithms described above, we measured the latency to target hole, the number and duration of nosepokes in each hole and the time spent in each of four quadrants over the testing period (Figures 3G–I). Video S3 demonstrates real-time tracking and scoring of nosepokes in a Barnes-maze experiment.

### Elevated zero-maze

The apparatus comprised of an elevated annular platform with two opposite, enclosed quadrants and two open quadrants. Mice were placed in the walled region and left undisturbed for 5 min. A user initialized the videos by identifying walled and open regions of the maze. In each frame, the software identified the mouse's centroid, area, and major axis length. We defined entry into the open regions when >95% of the mouse's area and its centroid were simultaneously in the open region. The amount of time spent in the open and walled regions was recorded as a measure of anxiety-like behavior (Jacobson et al., 2007) (Figures 3J,K). Since experimentally altered locomotion can influence the time spent in open or walled regions, independent of anxiety, we also measured ambulation. Risk assessment includes a stretch-attend posture in which the head extends into the open area but the remainder of the body stays in the walled compartment (Karlsson et al., 2005). This behavior was automatically identified when several empirical conditions were met: centroid of the mouse was in the walled region, head was in the open region, and the mouse's body length (major axis length of the segmented image) exceeded mean + 2^*^standard deviation of body length throughout the experiment.

### Rotarod performance

Animals were placed on a rotarod apparatus (model: ENV-577M, MedAssociates Inc., Georgia, VT) that accelerates linearly from 4 to 40 RPM over a 5-min session. Three trials, separated by an hour each, were conducted each day. Two measures were recorded for each rotarod test: the time lapsed until first fault, and the total time the animal remained on the rotating rod before falling. Fault was defined as making a complete revolution around the rotarod. In the event that an animal did not fault, we used fall time for fault.

### Fear conditioning

Contextual fear conditioning was performed as described previously (Bourtchuladze et al., 1994; Abel et al., 1997) to develop a complementary measure of hippocampal and amygdala function. On the training day, the mouse was placed in the conditioning chamber for 2:28 min before the onset of a foot shock (2-s 1.5 mA). Contextual conditioning was assessed 24 h later by placing the mouse back in the same chamber for 5 min. We implemented a simple yet robust algorithm to define periods where the animal stopped moving for at least 2 s, showing a “freezing” behavior that is traditionally recorded in fear conditioning tests. We used an image difference matrix, defined as the matrix created by subtracting an image at time *t_i_* with the preceding image at *t*_*i* − 1_. Theoretically, no motion between consecutive frames would yield a difference image matrix of all zeros. However, due to camera noise, a null image difference matrix rarely occurred. We estimated hardware noise by recording a 1-min video of an empty chamber, using consecutive image pairs and assigning a threshold motion limit (ε) equal to the 95th percentile of the matrix magnitude for image difference pairs. Freezing was designated to occur when consecutive image difference matrices over 2-s or longer duration (15+ image frame pairs) showed a net difference magnitude <ε (Figure 3L). A resulting bar code of activity (Figure 3L) denoted the periods of motion and inactivity over the 5-min monitoring period. Continuous scoring, rather than assessing freezing at arbitrary fixed time intervals, also permits analysis of cumulative freezing distributions. Video S4 demonstrates real-time scoring of freezing behavior.

### Validation and optimization of automated approaches

#### Comparison to manual scoring methods

We compared the results obtained from automated analysis to those obtained by manual scoring (visual inspection by an expert observer). In each task, we created a Bland-Altman plot to analyze the limits of agreement between the two methods (manual scoring being the gold standard). At least 20 videos each for fear conditioning, SOR, elevated-zero maze, and social interaction were manually scored. For each behavior task, we computed the mean and standard deviation of the difference between two values obtained by automated and manual scoring. Two expert observers scored the same videos to estimate inter-observer variability.

### Sensitivity analysis

#### Video quality

Videos were recorded in bright, even light conditions, using a high-definition camera. Segmentation by background subtraction was fast (<2 min for a 10-min video) and worked very well under these settings. To test its sensitivity to light conditions and video quality, we recorded a set of videos in lower resolution and in which the mouse was placed in an arena either dimly illuminated or not evenly illuminated.

#### Fear conditioning threshold

Assessment of freezing depends on estimating hardware noise; freezing was defined when the difference between successive frames drops below noise. Given a distribution of hardware noise obtained by recording a 1-min video of an empty chamber, we selected threshold values at the 50, 70, 90, and 95th percentile. We manually scored several experimental videos and compared the accuracy of the automated algorithm as a function of varying thresholds.

#### Interaction distance

In our implementation, interaction is scored by first defining a gaze vector originating from the nose and extending in the direction of vision with magnitude *x*. When this gaze vector crosses a user-defined ROI, it is scored as an interaction. To find the user-specific optimal magnitude of the gaze vector, users scored SOR videos frame-by-frame and annotated each frame with “interacting” or “not-interacting” labels. The same videos were processed with our algorithm. We swept through different magnitudes of the gaze vector (0–6″, step-size 0.1″) and for each vector length, we computed the total number of true positives and false positives. The user-specific interaction distance corresponds to the optimum point on the ROC curve, defined as the point on the ROC curve closest to the upper left corner (100% sensitivity and 100% specificity).

#### Statistical analysis

Statistical differences in task-related performance of animals in four experimental groups (WTLM sham, WTLM blast injured, Elk-1 KO sham, and Elk-1 KO blast) were assessed via One-Way ANOVA and Tukey's *post-hoc* test. Shapiro–Wilk test was used to assess normality and nonparametric tests (Kruskal–Wallis and Mann–Whitney U) were employed when needed. A repeated-measures (RM) ANOVA was performed when the same measurement was obtained for an animal over multiple trials as in rotarod or habituation. Group sizes were: WT sham *n* = 13, WT blast *n* = 13, Elk-1 KO sham *n* = 11, Elk-1 KO blast *n* = 12. alpha-level 0.05, ^*^p < 0.05 and ^**^p < 0.01 indicated significance. For a given level of analysis, a Bonferroni correction for multiple comparisons was used. All values reported are mean ± s.e.m. unless otherwise noted. Significance of time in all RM-ANOVA, *p* < 0.001 unless otherwise noted.

#### Behavior pattern analysis

The standardization of test scoring also provides an opportunity for employing a statistical framework for analyzing behavior patterns across experimental groups. Each animal was subjected to a battery of behavior tasks and 14 performance metrics were computed. Principal component analysis (PCA) visualized the dataset in a lower dimensional space and identified a combination of the original variables that explained the largest possible variation. Following PCA, a MANOVA identified a linear combination of the original variables with the largest separation between groups. Relationships between group means were visualized in a distance dendrogram. Additionally, the ability to use a pattern of behavior to correctly identify group membership was assessed by multiclass support vector machine (SVMlight; Joachims, 1999).

## Results

Our goal was to develop, assess, and apply an automated analysis of commonly used behavior tasks, including open field test, SOR, NOR, social interaction, Y-maze, Barnes maze, elevated zero-maze, and fear conditioning (Figures 2, 3). We used a subset of these tasks in this new toolbox and a systems-level analysis of behaviors tested to characterize a new transgenic mouse line (Elk-1 KO) and investigate the effects of bTBI on behavior.

### Comparison of automated and manual analysis of behavior tasks

**Figure 4:**
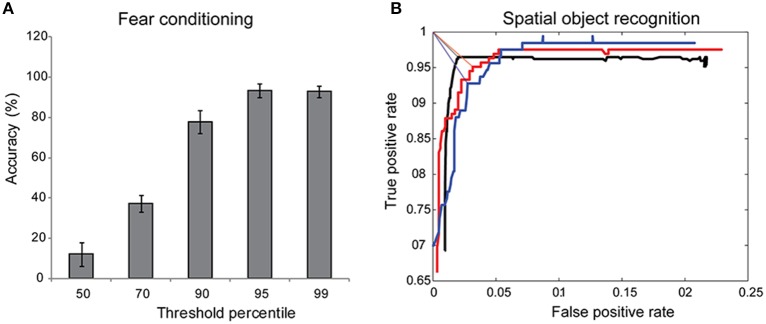
**Sensitivity of automated approach. (A)** Discriminating motion from freezing in automated scoring of fear conditioning experiments relies on choosing a threshold value for hardware noise. The accuracy of the automated approach compared to manual scoring approached >90% when point threshold value was at 95th percentile of hardware noise (*n* = 4). **(B)** Object interaction was defined when a gaze vector of magnitude *u* extending from the mouse's nose crossed a user-defined region of interest. This allowed us to calibrate the software to user's definition of interaction by determining the optimum *u* for each user. Three different users scored the same SOR video, annotating each frame in the video with “interacting” or “not-interacting” labels. An ROC curve generated by varying *u* identified the optimum interaction distance for each user as the point on the ROC curve closest to the upper left corner (true positive rate = 1, false positive rate = 0), denoted by straight lines.

To test whether our automated approach of discriminating motion from freezing was the ideal, we asked expert observers to score fear conditioning videos manually and compute total freeze fraction. We then computed the accuracy of automated method across a range of motion detection thresholds that corresponded to 50–99th percentile of the measure hardware noise. Across three independent scorers, we determined the optimal point hardware threshold corresponded to the 95th percentile of hardware noise (Figure 4A).

Assessment of social interaction, Y-maze, Barnes maze, SOR, and NOR all involve determining if an animal is interacting with a defined ROI. We expected slight variations on the definition of “interaction” for each person manually scoring the test. Existing proprietary software for automated analysis of these behavior tasks are closed box and either do not correctly identify the location of animal's head consistently or do not allow user flexibility in defining an interaction, resulting in gross over- or under-estimation of the true object interaction time. We used the automated tracking and gaze detection algorithm to examine different magnitudes of the gaze vector and determined the true positive rate and false positive rate for each vector length (Figure 4B), using the user definition of interaction as the gold standard. The optimal gaze distance was the vector length that minimized the distance from the upper left corner (perfect classification, TPR = 1, FPR = 0) on the ROC curve (Figure 4B). As expected, a single video analyzed by three different users produced three slightly different optimal vector lengths, reflecting the user-to-user variability in scoring interactions.

**Figure 5:**
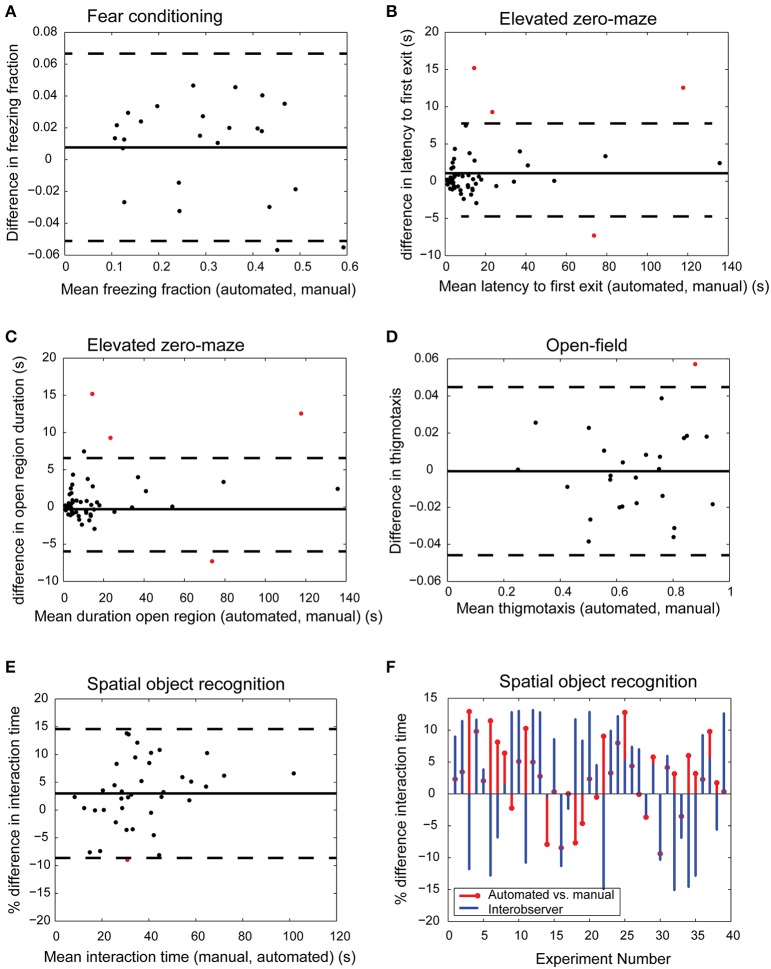
**Comparison of automated and manual scoring**. Bland-Altman plots show excellent agreement between manual and automated scores for freeze fraction in fear conditioning [**(A)** bias 5.24%, limits of agreement [−0.0511, 0.067] freeze fraction], latency to first exit [**(B)** bias 1.07-s, limits of agreement [−5.97 s, 8.11 s]], time spent in open region of the elevated zero maze [**(C)** bias −0.37 s, limits of agreement [−5.78 s, 5.04 s]], thigmotaxis in open-field experiment [**(D)** bias 0.003, limits of agreement [−0.046, 0.045]], and interaction time in spatial object recognition task [**(E)** bias 2.98%, limits of agreement [−8.62%, 14.6%]]. Bias and limits of agreement between automated and manual methods are denoted by horizontal solid and dashed lines in **(A–E)** (*n* ≥ 20 for each task). Red dots indicate measurements that fall outside limits of agreement. The difference in interaction time of automated and manual methods is comparable to inter-observer variability [**(F)** limits of agreement between automated and User A [−8.6%, 14.6%] and agreement between Users A and B [−17%, 21.8%]].

After confirming the robustness of our automated algorithms and calibrating them on a small subset of the recorded tests, we tested the accuracy of the automated video analysis in four specific behavior tasks: fear conditioning, SOR, elevated zero-maze, and open field test. Since social interaction and Barnes maze also require determining interaction with an ROI similar to SOR, we do not duplicate validation data here. For each task, 20 videos were both manually analyzed by trained observers and scored using the automated approach, resulting in 2 data points for each video. The mean biases of the automated approach relative to manual measurements were 5.24% for freezing time in fear conditioning task (Figure 5A), 1.07-s for latency to first-exit in elevated zero maze (Figure 5B), −0.37 s for amount of time spent in the open region in elevated zero maze (Figure 5C), 0.003 for thigmotaxis in open-field (Figure 5D), and 2.98% for object interaction time in SOR (Figure 5E).

We further tested the accuracy of automated scoring of interaction time using videos recorded in lower resolution (640 × 480 1″ = 23 pixels, high resolution 1200 × 1600 1″ = 57 pixels), dim lighting conditions, and uneven illumination. Segmentation via background subtraction was robust under dim and uneven lighting conditions. Lower resolution video footage was also adequate to accurately determine landmarks on the animal's body. The limits of agreement between automated and manual scoring across these three groups were comparable to videos acquired in high resolution under bright and even light conditions as in Figure 5E (low resolution: [0.4%, 6.1%], dim lighting: [−4.1%, 7.2%], uneven illumination: [−1.2%, 4.3%]).

Automated methods for assessing behavior not only increase throughput, but may potentially reduce user bias and variability. Forty SOR videos were manually scored for object interaction time in SOR experiments by two independent expert human observers, user A and user B. User A calibrated the automated approach using 3 videos chosen at random (Figure 4B). All videos were then automatically processed using the definition of interaction provided by User A. We compared the percent difference in interaction time between automated and User A, and between User A and User B (Figure 5F). The limits of agreement (bias ± 1.96^*^std) between automated and User A was [−8.62%, 14.6%], compared to [−17%, 21.8%] for User A vs. User B. The improved agreement between automated and User A is likely because User A calibrated the software to his/her own specification of interaction, yielding better agreement with the software than with another human observer.

Real-time tracking of the animal and scoring of object interaction is possible with our implementation. Our automated system consistently identified the correct coordinates of the nose and scored object interaction. There were few instances when the animal was sitting in a corner and in a curled posture where the algorithm did not correctly identify the head and tail coordinates. However, this did not pose a problem because objects are rarely placed in the corners and mislabeled events span less than 2–3 consecutive frames. Additionally, since each video frame is automatically annotated with “interacting” or “not-interacting” labels, we were able to quickly scroll through a set of interacting frames and remove false positives. In our experience, manual correction took less than 1 min for a 10 min video and improved the sensitivity to nearly 98%.

### Autotyping as a method to assess the influence of blast-injury and Elk-1 deletion

With these validated algorithms for automating the analysis of individual behavioral tasks, we examined if bTBI caused a significant change in the normal behavior of C57/BL6Nwildtype mice. In addition, we explored if there were significant behavioral differences that appeared when a neuronal transcription factor, Elk-1, was deleted completely from a C57/BL6N animal background and whether behavioral impairments following bTBI can be ameliorated with Elk-1 deletion. Several recent reports implicate Elk-1 in neuronal loss and degeneration (Sharma et al., 2010; Morris et al., 2013), however it is unclear if (a) Elk-1 is important for normal behavior and (b) whether Elk-1 deletion improves outcome after bTBI.

PCR confirmed the deletion of Elk-1 in KO male animals, and littermate wildtype animals retained Elk-1 mRNA levels similar to native wildtype (data not shown). Animals placed in an open field environment, subject to elevated zero maze testing, and exposed to SOR and fear conditioning testing over an eight day interval showed no significant differences between littermate wildtype and KO groups using ANOVA testing. The lack of an overt behavioral phenotype is not surprising, given the compensatory pathways available for other isoforms of the Elk-1 protein not affected by the KO strategy employed (Cesari et al., 2004).

We next applied our analysis to examine if bTBI caused a significant change in the normal behavior, and if these changes were influenced by the deletion of Elk-1. Studying a range of behavioral tasks, rather than a single task, is particularly important because of the widespread changes that can occur throughout the brain following a gene deletion and bTBI alike (Davenport et al., 2012). We focused our behavior analysis on specific tests that relate to deficits appearing in patients following blast-induced TBI, including memory deficits, heightened anxiety, concentration difficulty, and balance problems. Therefore, we selected the rotarod, elevated zero maze, open field, SOR, and fear conditioning tests to explore the deficits appearing after blast exposure, and how these deficits changed in Elk-1 KO animals.

### Blast-injury increases generalized anxiety in wildtype animals while Elk-1 knockout mice are resistant to post-blast anxiety

**Figure 6:**
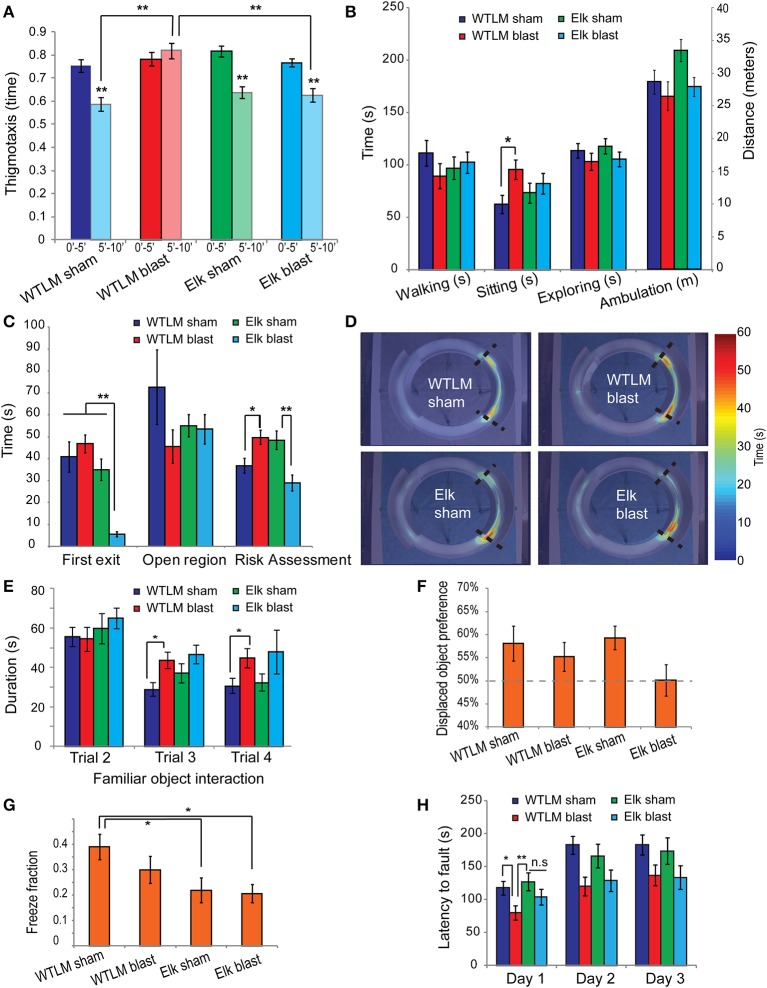
**Behavior deficits following bTBI in wildtype littermate and Elk-1 knockout mice. (A,B)** Open-field. **(A)** Thigmotaxis decreased from the first 5-min interval to the second 5-min interval in wildtype sham (paired *t*-test *p* < 0.001, *n* = 13), Elk-1 KO sham (*p* < 0.001, *n* = 11) and Elk-1 KO blast injured animals (*p* < 0.001, *n* = 12) but was not significantly different in wildtype bTBI (*p* = 0.194, *n* = 12). **(B)** Wildtype bTBI animals spent significantly more time sitting in the open-field compared to uninjured shams (*p* = 0.0484). Other open-field measures were not different across the four groups (ANOVA *p* > 0.05). **(C–D)** Elevated zero-maze. **(C)** Latency to first exit of walled regions and risk assessment was significantly lower in Elk-1 KO bTBI compared to Elk-1 KO sham (*p* < 0.01). However risk assessment was significantly elevated in wildtype bTBI relative to sham (*p* = 0.0312). **(D)** Average heat-map showed an increased localization to the walled/open interface in wildtype bTBI group. **(E–F)** Spatial object recognition. **(E)** Object habituation was significantly impaired in wildtype bTBI compared to sham (RM-ANOVA *p* < 0.005) but was not different between Elk-1 KO sham and injured animals (*p* = 0.181) **(F)** Preference for the displaced object was >50% for wildtype sham, blast and Elk-1 KO sham groups suggesting acquisition of spatial memory. However, displaced object preference was reduced in blast injured Elk-1 KO (50.1 ± 3.4% Elk+blast vs. 59.3 ± 2.6% Elk+sham, *p* = 0.0531). **(G)** Elk-1 KO sham showed a deficit in fear conditioning compared to wildtype sham (*p* = 0.0213) and thisimpairment was not worsened by bTBI (*p* > 0.05). **(H)** Motor coordination and motor memory was assessed by computing latency to fault on rotarod. On day 1, WTLM blast had significantly lower fault time compared to both WTLM sham and Elk-1 KO sham (WT blast 79.8 s ± 10.8 s vs. sham 117.9 s ± 10.5 s, *p* = 0.0145; WT blast vs. Elk-1 sham 127.3 s ± 13.5 s, *p* = 0.0074). An improvement in fault was observed over days 1–3 for all four groups, however, the improvement was greater for uninjured shams than injured animals, regardless of genotype (repeated-measures ANOVA within subjects time *p* < 0.001, between subjects sham vs. blast *p* = 0.0037, wildtype vs. KO *p* = 0.8712). ^*^p < 0.05, ^**^p < 0.01.

Our collective results from open-field and elevated zero-maze tests show that bTBI significantly increases anxiety-like behavior. Uninjured animals placed in an open-field arena showed a typical spatiotemporal response to novel environment, spending most of their time along the periphery (thigmotaxis) during the first 5 min and gradually entering the central zone of the arena during the next two 5 min intervals. We quantified thigmotaxis by determining the ratio of time spent along the periphery relative to time spent in the center over any 5-min interval as an index of anxiety (Simon et al., 1994). Following bTBI, wildtype animals show increased thigmotaxis during the second 5 min interval compared to sham group (mean ± s.e.m.: 0.820 ± 0.033 blast vs. 0.588 ± 0.039 sham, *p* = 0.0013, Figure 6A). In addition, blast injured mice spent significantly more time sitting in an open-field arena compared to uninjured shams, another measure of anxiety (Prut and Belzung, 2003) (95.81 s ± 9.19 s blast vs. 62.56 s ± 8.83 s sham, *p* = 0.0484, Figure 6B). The total distance traveled and time spent walking or exploring were not significantly different between sham and injured wildtype animals, suggesting that the spatial component important in thigmotactic behavior is being directly increased by blast.

In contrast to WTLMs, blast-injured Elk-1 KO animals did not show a significant difference in thigmotaxis or total time spent sitting compared to uninjured sham Elk-1 KO controls (thigmotaxis: 0.626 ± 0.028 blast vs. 0.638 ±0.026 sham, *p* > 0.05; sitting: 82.3 s ± 9.69 s blast vs. 73.4 s ± 9.82 s sham, *p* > 0.05). Moreover, blast-injury in Elk-1 KO group resulted in significantly less thigmotaxis compared to blast injured WTLM, suggesting a possible role for Elk-1 in post-traumatic anxiety (0.626 ± 0.028 Elk+blast vs. 0.820 ± 0.033 WTLM+blast, *p* = 0.0081).

An alternative test for anxiety-like behavior is the elevated zero maze. Indicators of increased anxiety include a relative increase in latency to first exit, decreased time spent in the open unprotected region, and increased risk assessment behaviors. We found increased risk assessment activity in WTLM blast group relative to uninjured sham (49.8 s ± 4.08 s blast vs. 36.8 s ± 3.41 s sham, *p* = 0.0312, Figure 6C). No significant difference was found between WTLM blast and WTLM sham groups in latency to first exit or time spent in unprotected open regions (Figure 6C). We observed a very significant decrease in latency to first exit in Elk-1 KO blast injured mice relative to 3 other groups (5.63 s ± 1.14 s Elk+blast vs. 40.82 s ± 6.87 s WTLM sham, 46.8 s ±4.08 s WTLM blast, 35 s ± 4.9 s Elk sham, *p* < 0.001, Figure 6C). Similar to decreased latencies to exit, a decrease in risk assessment behavior appeared in Elk-1 KO blast injured mice (Figures 6C,D). The cumulative distance traveled in the zero-maze, as well as the peak instantaneous speed, were not statistically different between the 4 groups (ANOVA, *p* > 0.05, data not shown).

The behavioral alterations of animals using two anxiety-related assessments, open-field test and elevated zero-maze indicate heightened anxiety following blast-injury in WTLM. In contrast, blast-injury does not worsen anxiety-related behavior in Elk-1 KO mice relative to their sham counterparts.

### Blast-injury to wildtype mice impairs object habituation but Elk-1 deletion recovers normal behavior

Habituation is one form of nonassociative learning that can be readily measured in the SOR test where exploration of the objects during consecutive training trials decreases as novelty decreases (i.e., before one of the objects is displaced). Therefore, we analyzed the duration of interaction with the non-displaced object in trials 2–4 of the SOR test in mice that received bTBI prior to training. Uninjured wildtype sham mice habituate to the SOR arena as the duration of interaction with the non-displaced object significantly decreased over time (RM-ANOVA, *p* = 0.0062, Figure 6E). In contrast, blast injured wildtype animals failed to show a significant decline in object exploration from trial 2 to trials 3 and 4 (RM-ANOVA *p* > 0.05). Direct comparison between sham and blast injured wildtype animals showed a significant deficit in object habituation during trial 3 (blast: 42.8 s ± 4.12 s, sham: 26.1 s ± 5.03 s, *p* = 0.0036).

In contrast to WTLM, blast injured Elk-1 KO animals did not show a deficit in object habituation compared to sham (multivariate RM-ANOVA, *p* > 0.05). Both sham and injured Elk-1 KO groups spent equally large amounts of time interacting with the non-displaced object in trial 2 (first exposure to objects in the arena) and significantly less time in trials 3 and 4 (Trial 3: Elk-1 KO sham, 37.1 s ± 2.36 s compared to Elk-KO injured, 46.6 s ± 2.26 s, *p* = 0.2366).

### Blast injury impairs spatial and associative memory only in Elk-1 knockout mice

We assessed spatial memory by calculating the percent of total object interaction time that was devoted to the displaced object in the SOR test during trial 5. Typically, by trial 4, mice spend nearly equal time interacting with the two objects (Supplementary Figure 1B). Upon displacing an object in trial 5, both wildtype sham and blast injured animals spent significantly more time (>50%) interacting with the displaced object, consistent with acquisition of spatial memory. Preference for the displaced-object was not different between sham and injured wildtype animals (wildtype sham 58.1 ± 3.8% vs. wildtype injured 55.2 ± 3.2%, *p* > 0.05). Similarly, Elk-1 KO sham animals showed a preference for the displaced object in trial 5. However, the preference for displaced object was abolished in blast injured Elk-1 KO group (Elk-1 KO sham 59.3 ± 2.6% vs. Elk-1 KO injured 50.1 ± 3.4%, *p* = 0.0034) (Figure 6F).

Since blast injured WT animals still retained spatial memory, we next tested contextual fear memory, a distinct hippocampus-dependent form of associative memory. Pairing of an aversive foot shock to a novel environment resulted in freezing responses when mice were reintroduced to the same environment 24-h following the shock. We found no statistical difference in total freeze fraction between sham and blast injured wildtype animals (sham: 0.390 ± 0.049, 0.3 ± 0.053, *p* = 0.18) suggesting that associative memory is not altered following blast-injury (Figure 6G).

Unlike wildtype mice, Elk-1 KO showed significantly less freezing behavior (wildtype sham freeze fraction: 0.3904 ± 0.0494, Elk-1 KO sham: 0.2198 ± 0.0492, *p* = 0.0213). However, the impairment in associative memory was not made worse by blast-injury (Elk-1 KO blast: 0.2069 ± 0.035, *p* > 0.05 compared to Elk-1 KO sham) (Figure 6G). A deficit in contextual fear conditioning in Elk-1 KO mice suggests an important role for this transcription factor in associative memory. Indeed, this is consistent with a previous report of increased Elk-1 phosphorylation in the CA3 hippocampus and dentate gyrus following contextual fear conditioning and the proposed role of Elk-1 in consolidation of contextual memories via interaction with Erk1/2 proteins (Sananbenesi et al., 2002).

### Blast-injury impairs motor coordination and motor learning

We assessed motor coordination and motor learning in rotarod task by measuring the latency to fault. On first exposure to the rotarod (day 1), wildtype blast injured animals had significantly lower fault time compared to wildtype sham, suggesting a deficit in motor coordination as a result of blast (wildtype blast fault 79.8 s ± 10.8 s vs. wildtype sham 117.9 s ± 10.5 s, *p* = 0.0145) (Figure 6H). Interestingly, Elk-1 KO animals were resistant to blast-induced deficits in motor coordination (Elk sham fault: 127.3 s ± 13.5 vs. Elk blast fault: 104.2 ± 12.2, *p* = 0.2097).

An improved performance on the rotarod during subsequent trials 2 and 3 is indicative of acquisition of motor memory. All four groups showed an improvement in latency to fault over days 1–3, but the increase in performance was greater for uninjured shams than blast-injured animals regardless of genotype (RM-ANOVA, within subjects time *p* < 0.0001, between subjects sham vs. blast *p* = 0.0037, wildtype vs. KO *p* = 0.8712). Together, blast-injury impairs the acquisition of motor memory in WTLMs and Elk-1 KO mice equally.

### Multivariate analysis reveals the relative effects of genotype, injury, and genotype^*^injury on behavior outcome

An automated approach permits the measurement of even more behavioral responses in a high-throughput fashion. With the goal of automating the process of phenotyping animal behavior, we also sought to determine whether there are group differences when the aggregate behavior was considered simultaneously, rather than individually across each behavior test. Rather than comparing group means on a single variable (as in Figure 6), we now compared group centroids for the 14 variables collected across the 4 independent behavior tests.

**Figure 7:**
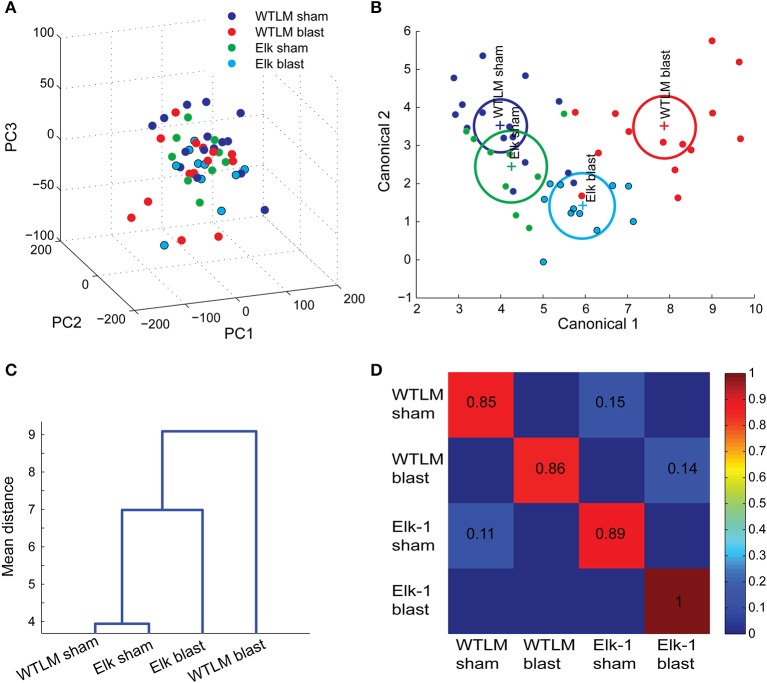
Multivariate analysis reveals the relative effects of genotype, injury, and genotype^*^injury on behavior outcome. (A) Projection of 14 behavior attributes for each animal onto the first three principal components did not reveal obvious groupings. **(B)** Multivariate ANOVA identified differences in the population means of the four groups (Wilks' λ = 0.0011). The multivariate behavior scores are projected onto a MANOVA canonical subspace and color-coded by experimental groups (dots represent the aggregate neurobehavior of individual mice, + marker indicates group centroids with 95% confidence bounds shown in circles). **(C)** Dendrogram of pair-wise group centroids reveals the hierarchical similarity among groups. **(D)** Confusion matrix. A multiclass support vector machine was trained using multivariate behaviors to determine whether a pattern of task-related behaviors can accurately predict injury severity or genotype. The fraction of a group of mice (along the rows) that were classified as each of the four alternative groups (along the columns) are indicated in the confusion matrix.

With the large number of behavior measurements, we first applied PCA for clustering and exploratory analysis. Visualizing the behavior dataset in a subspace spanned by the first three principal components (Figure 7A, 72% explained variability) does not show a natural clustering of mice into separate groups. An alternative approach using MANOVA was used to identify a linear combination of the original behavior variables with the largest separation between groups. Response variables with pair-wise correlation greater than 0.7 were eliminated from MANOVA design to avoid over-bias in the analysis (Supplementary Figure 1A). All variables used in the MANOVA (see Supplementary text for tabular listing) followed a multivariate normal distribution and had equal variances (Barlett's test, *p* > 0.1, n.s.). We found a significant difference in overall group mean centroids, Wilk's lambda *p* = 0.0011. Genotype alone did not have an effect on multivariate group mean differences (WTLM vs. Elk-1 KO, *p* = 0.0825), however, injury severity (sham vs. blast, *p* = 0.0007) and genotype^*^injury (*p* = 0.0018) were both significant. We projected these multivariate behavior scores for each mouse onto a canonical subspace and color-coded each group (Figure 7B). Inspection of the group mean centroids (+ marker) and 95% confidence bounds reveals intersecting groups with no significant difference from each other (WTLM sham vs. Elk sham), while non-intersecting domains represent groups that are significantly different from each other (e.g., Elk-1 KO sham vs. Elk-1 KO injured). Using this canonical representation, a dendrogram constructed from pair-wise Mahalanobis distances between each pair of group means identified the hierarchical similarity among groups—WTLM sham and Elk-1 KO sham were phenotypically most similar; blast injury affects the two genotypes differently—wild-type injured mice are most affected while Elk-1 KO injured have milder phenotypic alterations (Figure 7C).

Until now, we relied only on retrospective data mining to group aggregate behaviors. With the ability to quickly screen several tasks simultaneously, there is an opportunity to use these behavior data as prognostics. In this light, we tested whether pattern of task-related neurobehavior can accurately predict the injury severity or genotype of an animal. We trained and tested a linear multiclass support vector machine using the 14 behavior attributes. The results of a leave-one-animal-out cross validation are shown in a confusion matrix (Figure 7D). The confusion matrix indicates the fraction of a group of mice (along the rows) that were classified, on the basis of its pattern of behavior, as each of the four alternative groups (along the columns). Larger values along the diagonal indicate successful classification. As expected, the classification accuracy for wild-type sham and blast injured groups is the largest, while there is large confusion in accurately classifying animals into WTLM sham and Elk-1 KO sham groups—only 40% of true Elk-1 KO sham animals were correctly classified as Elk-1 KO sham, while 30% were falsely classified as WTLM sham.

## Discussion

We identified and incorporated a number of automation algorithms to generate a new, open access software platform for scoring and analyzing several common behavioral tasks. Automated scoring can be done in real-time and the results matched manual measurements within the limits of inter-observer variability. We then applied automated tools to phenotype animals carrying a genetic manipulation (Elk-1 KO), experimental manipulation (blast TBI), and the combination of these two effects. Examining the behaviors separately, we discovered that blast-injury significantly increased the level of anxiety and impaired the ability to habituate to a novel environment. Elk-1 KO animals were resistant to these detrimental effects of blast-injury, but showed a deficit in associative memory after blast exposure. A multivariate analysis designed to identify differences in aggregate behavior showed that Elk-1 KO and wildtype animals were not significantly different prior to blast-injury. Following injury, wildtype animals showed more severe changes in behavior than Elk-1 KO animals.

Our application of the software toolkit to evaluate the pattern of deficits appearing following blast-induced brain injury provides a new, more comprehensive view of the deficits caused by blast exposure. Blast-injury is characterized by modest neuronal loss or pathologic remodeling that can disrupt both anatomic and functional connectivity throughout the brain (Levin et al., 2010; Sponheim et al., 2011; Magnuson et al., 2012; Mac Donald et al., 2013). Given this potential broad disruption of brain networks, our automated screening tool was an ideal method to scan across multiple behavior tasks and develop a behavioral phenotype for each animal. The early signs of anxiety observed in our wildtype mice are reminiscent of symptoms associated with post-traumatic stress disorder in human blast TBI, and is consistent with some evidence from other rodent models of bTBI (Park et al., 2013). At the level of blast exposure studied, we saw no significant memory deficits using two independent measures of associative learning—contextual fear conditioning, and SOR. However, we found a significant reduction in motor memory following blast. The consistent appearance of a memory deficit is not a universal consequence of bTBI in rodents, and some of these deficits appear to be linked to the head accelerations induced by the blast exposure (Goldstein et al., 2012).

To our knowledge, this work also presents the first evidence that Elk-1 plays an important role in the recovery of function after a neurological injury. One key modulatory point for controlling the function of Elk-1 is its multisite phosphorylation “state.” The mitogen activated protein kinase ERK phosphorylates Elk-1 on multiple sites, and the ERK pathway is activated in several models of TBI (Otani et al., 2002; Carbonell and Mandell, 2003; Raghupathi et al., 2003). However, many of the controlling phosphatases and kinases regulating the control of Elk-1 within its transactivation domain (Yang et al., 2002), as well as the domain controlling its neurodegenerative function (Barrett et al., 2006; Sharma et al., 2010) are not known. Based on our current data, we cannot conclude if the behavioral differences between Elk-1 KO and WTLMs is simply because the KO animals have lost the ability to prune dysfunctional neurons from hippocampal and cortical circuits, or if these changes are more linked to Elk-1 dependent changes in gene expression. Determining the key regulating mechanisms that mediate these Elk-1 dependent effects is particularly important because we found that Elk-1 deletion can eliminate posttraumatic anxiety. Given that posttraumatic stress disorder is a condition commonly associated with soldiers exposed to blast, a more thorough exploration of these Elk-1 dependent mechanisms of anxiogenic behavior may yield important insights for a significant clinical condition.

From a broader perspective, the rapid scanning of several behaviors in parallel facilitates a new framework to assess the broad effects that can occur in a rodent model of neurological disease. Compared to manual scoring, our automated analysis can reduce user-to-user variability or observer bias. This leads to more consistent findings within and across laboratories. Further, an automated method greatly speeds up data analysis and lessens the time burden on researchers, making more complex behavior protocols possible. We expect the broader behavior spectrum that can be analyzed with our autotyping system will permit a more complete and rapid understanding of disease models in rodents, with the goal of using this same toolbox to test potential treatment strategies.

## Author contributions

Tapan P. Patel and David F. Meaney conceived of the idea and wrote the manuscript. Tapan P. Patel implemented the algorithms, analyzed videos, and the resulting data. David M. Gullotti performed the animal experiments. All authors were involved in the data interpretation, experimental design, and the discussions in the selection of the neurobehavior measures. All authors contributed to the editing of the manuscript.

### Conflict of interest statement

The authors declare that the research was conducted in the absence of any commercial or financial relationships that could be construed as a potential conflict of interest.

## Supplementary material

MATLAB implementation and user guide are available at: http://www.seas.upenn.edu/~molneuro/autotyping.html

Video S1 **Scoring of object interaction**. Overview of spatial object recognition scoring module, also applicable for other “interaction” tasks. Typically, a video recording contains multiple enclosed boxes and each box may contain variable number of objects in a particular spatial configuration. Using an initialization GUI, users define the number of boxes and the number of objects per box for an experiment and interactively define regions of interest. Once several videos are initialized, scoring is done as a batch job. An example of real-time tracking of mice is illustrated. **Top panel:** The centroid and head of the animal are automatically detected in each frame and marked in green and red dots. A vector in the direction of the animal's gaze is marked in red (vector magnitude increased for visual clarity). Interaction is scored when the gaze vector crosses a user-defined region of interest (glass and metal objects). The boundary of interacting object becomes highlighted in red during the movie. **Bottom panel:** The cumulative time spent in the arena during each bout of interaction. Movie is sped-up x3. Once videos are scored, users can quickly scroll through a set of frames labeled as interacting and verify the accuracy of the algorithm or remove any false-positives if needed.

Video S2 **Performance in Y-maze**. Overview of YMaze scoring module, also applicable for other maze-like configurations, including T-maze, zero-maze, open field, place preference, etc. Separate GUIs and modules for each are provided in the toolbox. Users define regions of interest, such as arms of a maze or subdivisions of an arena and the algorithm computes the amount of time spent in each ROI and the number of transitions between the ROIs. A simple visual output in a bar-code like format is generated for each experiment, which can be useful to detect patterns of exploration. Common measures of performance specific to different maze-like configurations, such as latency to first exit and risk assessment in elevated zero-maze or path length and number of errors for Barnes maze are computed.

Video S3 **Performance in Barnes-maze**. Real-time tracking of mouse's location, the number and duration of nosepokes in a Barnes-maze is illustrated. The coordinates of the mouse's nose are determined, as outlined in Methods. A nosepoke is defined when the coordinates of the animal's nose cross a circular hole and is highlighted by a red outline in the video.

Video S4 **Scoring of fear conditioning**. Detection of freezing events in a fear conditioning chamber. Red border around a video frame indicates freezing bout.
