# Supplementary material for: An open-source toolbox for automated phenotyping of mice in behavioral tasks
Source: Front Behav Neurosci. 2014 Oct 8;8:349. doi: 10.3389/fnbeh.2014.00349 (PMC4189437; doi:10.3389/fnbeh.2014.00349)
Supplement: Supplementary file 5 [file DataSheet1.DOCX]

**Supplementary Figure 1:**

**
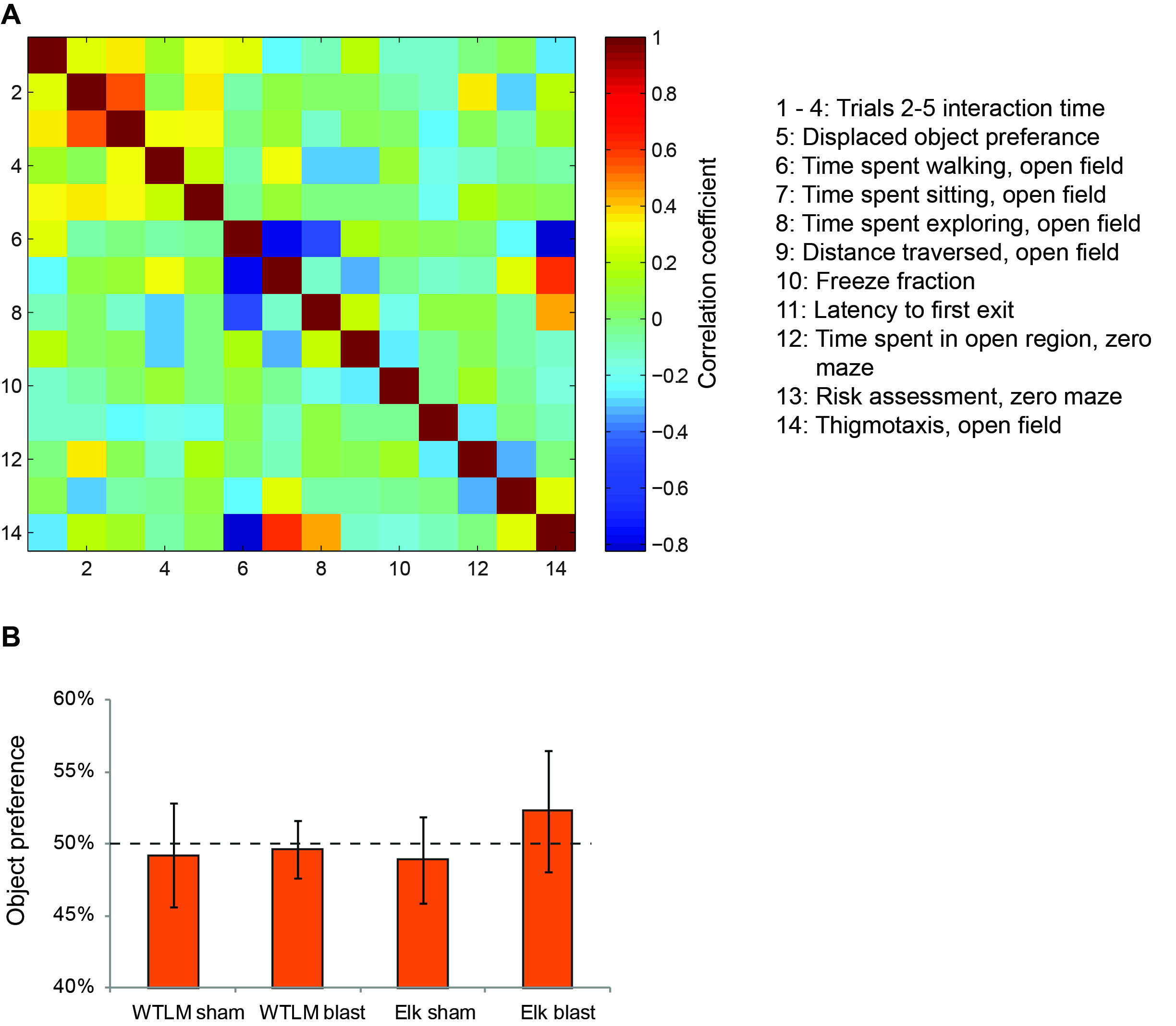
**

**Supplemental Figure 1. A**: Pair-wise correlation matrix of the full dataset consisting of 47 animals spread across 4 groups and 14 behavior variables revealed strong negative correlation (absolute correlation coefficient > 0.7) between time spent walking in the open-field test (S1A, row 6) and time spent sitting (row 7) and thigmotaxis (row 14). The 'time spent walking' variable was excluded in MANOVA. All variables followed a multivariate normal distribution and had equal variances (Barlett's test, *p*>0.1). **B:** In spatial object recognition task, mice are exposed to an arena containing 2 objects (glass bottle and metal cylinder) for three 10-minute training sessions. One of the objects is then displaced in test session. Preference for object A over object B, defined as the fraction of total interaction time devoted to object A, was nearly 50% for all 4 groups at the end of the last training session.
